# The Why of YY1: Mechanisms of Transcriptional Regulation by Yin Yang 1

**DOI:** 10.3389/fcell.2020.592164

**Published:** 2020-09-30

**Authors:** Thijs C. J. Verheul, Levi van Hijfte, Elena Perenthaler, Tahsin Stefan Barakat

**Affiliations:** ^1^Department of Cell Biology, Erasmus MC University Medical Center, Rotterdam, Netherlands; ^2^Department of Neurology, Erasmus MC University Medical Center, Rotterdam, Netherlands; ^3^Department of Clinical Genetics, Erasmus MC University Medical Center, Rotterdam, Netherlands

**Keywords:** YY1, neurodevelopment, gene regulation, non-coding genome, enhancer, non-coding regulatory element

## Abstract

First described in 1991, Yin Yang 1 (YY1) is a transcription factor that is ubiquitously expressed throughout mammalian cells. It regulates both transcriptional activation and repression, in a seemingly context-dependent manner. YY1 has a well-established role in the development of the central nervous system, where it is involved in neurogenesis and maintenance of homeostasis in the developing brain. In neurodevelopmental and neurodegenerative disease, the crucial role of YY1 in cellular processes in the central nervous system is further underscored. In this mini-review, we discuss the various mechanisms leading to the transcriptional activating and repressing roles of YY1, including its role as a traditional transcription factor, its interactions with cofactors and chromatin modifiers, the role of YY1 in the non-coding genome and 3D chromatin organization and the possible implications of the phase-separation mechanism on YY1 function. We provide examples on how these processes can be involved in normal development and how alterations can lead to various diseases.

## Introduction

Yin Yang 1 (YY1) is a transcription factor (TF) that was first described in 1991 by three independent groups, who all named the protein differently based on the molecular mechanisms they associated it with. It was called NF-E1, as it binds the μE1 intron enhancer at the immunoglobulin heavy chain (IgH) locus (Park and Atchison, 1991) or δ, as it binds the delta motif in the promoter of ribosomal protein genes (Hariharan et al., 1991). The name Yin Yang 1 was eventually broadly adopted, as it captures the dual activity of YY1 as both a transcriptional activator and repressor (Shi et al., 1991). Similarly, the YY1 family member YY2 has this dual activity (Nguyen et al., 2004).

**FIGURE 1:**
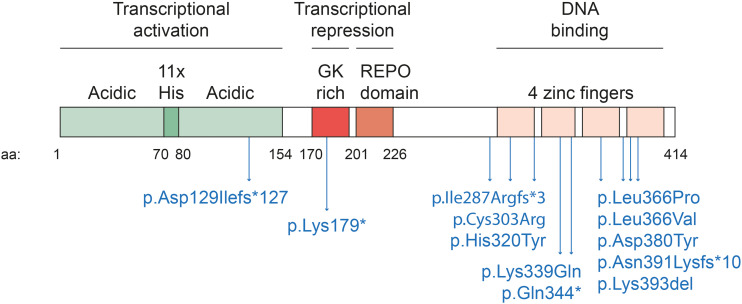
Schematic diagram of human YY1 domains. Human YY1 is composed of 414 amino acids. It binds a small DNA sequence (5′-CCGCCATNTT-3′) through the four C2H2-type zinc fingers located at the C-terminal of the protein (amino acid 296–320, 325–347, 353–377, 383–407). The REPO domain (aa 201–226) and a glycine-lysine rich domain (GK-rich, aa 170–200) mediate transcriptional repression. The REPO domain is responsible for the interaction with polycomb group proteins while the GK rich domain with histone deacetylases (HDAC). The N-terminal region of the protein mediates transcriptional activation. It is composed mainly by acidic amino acids (aa 1–154) and by a stretch of 11 histidines (aa 70–80), that are thought to stimulate YY1 accumulation in nuclear speckles. In blue, the reported causative variants of Gabriele-de Vries syndrome are indicated.

YY1 is ubiquitously expressed in mammalian cells. It forms homodimers that seem to be stabilized by low specificity RNA binding (Wai et al., 2016). YY1 homodimers bind a relatively small sequence motif (5′-CCGCCATNTT-3′), often found in enhancers and promoters, via the four C2H2 zinc fingers in its C-terminal domain (Hyde-DeRuyscher et al., 1995; Yant et al., 1995; Figure 1). YY1 and its paralog, YY2, share overall 56% homology at the aminoacid sequence, reaching 86% of conservation in the zinc-fingers region. This high degree of homology might result in YY2 binding to the same YY1 DNA binding motif (Nguyen et al., 2004). The DNA binding zinc fingers partially overlap with sequences involved in transcriptional repression. In the N-terminal region a transcriptional activator domain is located (Shi et al., 1991). YY1 influences transcription by the recruitment of cofactors (Gordon et al., 2006). For example, the REPO domain interacts with polycomb group proteins, to recruit repressive cofactors to specific genes (Wilkinson et al., 2006, 2010). Cohesin and condensin, key factors of 3D chromatin organization, also interact with the REPO domain of YY1 (Pan et al., 2013). Another YY1 domain contains a his tract consisting of eleven consecutive histidine residues, and is proposed to stimulate YY1 accumulation in nuclear speckles (Salichs et al., 2009), that play an important role in RNA metabolism (Galganski et al., 2017). Interestingly, YY1 itself plays a role in pre-mRNA splicing (Rambout et al., 2018). Binding intronic enhancer motives allows YY1 to activate gene expression and promote splicing (Bianchi et al., 2013).

YY1 is a crucial factor regulating cell proliferation and apoptosis (Deng et al., 2010; Nicholson et al., 2011) and it has been implicated in many regulatory mechanisms in different tissues. In the brain, YY1 plays a well-established role in neuronal development (Gabriele et al., 2017; Morales-Rosado et al., 2018; Zurkirchen et al., 2019). In mice, a homozygous *Yy1* knock-out results in peri-implantation lethality while heterozygous mutations cause growth retardation and neurulation defects (Donohoe et al., 1999). In humans, YY1 haploinsufficiency causes Gabriele-de Vries syndrome (OMIM **#**617557) (Gabriele et al., 2017; Morales-Rosado et al., 2018), characterized by psychomotor delay and intellectual disability alongside many comorbidities, including craniofacial dysmorphisms, intra-uterine growth restriction and behavioral alterations.

Recent findings concerning YY1 activity in chromatin rearrangement and its importance in neuronal development have caused re-evaluation of YY1 function. This review focusses on basic transcriptional mechanisms that YY1 has been implicated in and considers its function in context of neuronal development and disease.

## Mechanisms of Gene Activation and Silencing by YY1

### The Function of YY1 as a Traditional DNA-Binding Transcription Factor

A key question in the YY1 field is how a DNA-binding TF can act as both a transcriptional activator and repressor. Multiple mechanisms have been suggested, including post-translational modifications of YY1 (Yao et al., 2001) and co-factor dependency (Gordon et al., 2006). Acetylation of the C-terminal domain of YY1, for example, reduces DNA-binding capacity *in vitro*, whereas acetylation of the central domain is required for YY1 to act as a full repressor (Yao et al., 2001). In mouse anterior neuroectoderm development, the acetylation of YY1 is required for *Otx2* activation (Takasaki et al., 2007). Only acetylated YY1 binds an essential enhancer 5kb upstream of the homeobox gene *Otx2* to prompt its expression (Takasaki et al., 2007). Post-translational modifications of YY1, thus, seem to influence whether YY1 can activate or repress transcription.

Another explanation for YY1s context-dependent transcriptional activation or repression is the interplay between YY1 and its cofactors (Yao et al., 2001). Protein-protein interactions exist between YY1 and DNA-binding cofactors such as SpI (Seto et al., 1993) and c-Myc (Shrivastava et al., 1993). Bound to DNA, YY1 tethers cofactors directly to promoters, such as YY1 associated factor 1 (YY1AP), that boosts transcription (Wang et al., 2004). YARP, a structural homolog of YY1AP, is expressed in brain, heart and placenta tissue suggesting it acts as another co-activator of YY1 (Ohtomo et al., 2007). Additionally, YY1 also recruits co-repressors to the DNA, such as Smad family members repressing TGF-β signaling and cell differentiation (Kurisaki et al., 2003). *In vitro*, YY1 is able to bind promoter sequences and recruit polymerase by interacting with general TFs. Overlapping DNA binding sites of YY1 and transcriptional activators provide another explanation for YY1-mediated repression. Since DNA-binding of YY1 and an activator at a given locus can be mutually exclusive, YY1 binding can block the activating factor. For example, in mammary epithelial cells, YY1 represses β-casein competing with the latent-state mammary gland specific factor (MGF) of the STAT family, STAT5A (Raught et al., 1994). Upon lactation, MGF is activated, increasing its DNA-binding affinity and enabling it to replace YY1 and de-repress β-casein (Shi et al., 1997). Independent of binding the DNA, YY1 can regulate transcription by protein-protein interactions with other regulators (Galvin and Shi, 1997). In mouse neurons, Yy1 and its interacting partner Brd4 activate Senp1, an upstream regulator of glutamate signaling, which plays a pivotal role in neuronal plasticity (Wu and Donohoe, 2019). Dephosphorylation of Yy1 upon membrane depolarization depletes the *Senp1* promoter of Yy1-Brd4, consequently repressing Senp1 expression (Wu and Donohoe, 2019). This example illustrates the mechanisms of both post-translational YY1 modifications and co-factor dependency that partly explain how a ubiquitously expressed TF can be an activator or repressor depending on the cellular context.

### The Interplay Between YY1 and Chromatin Modifications

Among YY1 interactors, are multiple chromatin modifiers, suggesting chromatin modifications might explain YY1 functioning as a transcriptional repressor or activator. YY1 can direct the Polycomb complex to specific DNA loci, initiating methylation of histone 3 lysine 27 (H3K27me3) (Atchison et al., 2003; Wilkinson et al., 2006). Furthermore, YY1 interacts with histone deacetylases (HDACs) associated with gene silencing. Several members of this family, such as HDAC1, HDAC2 and HDAC3, interact with YY1 both *in vitro* and *in vivo* (Yang et al., 1997). Interestingly, YY1 also interacts with histone acetyltransferases (HATs) like p300 (Lee et al., 1995) and CREB binding protein (CBP), activating transcription (Lee et al., 1995; Austen et al., 1997). Besides acetylating or de-acetylating histones, HATs and HDACs modify YY1 itself, regulating its DNA-binding affinity and activity as a transcriptional regulator (Yao et al., 2001). In addition to histone 3 modifications, YY1 promotes transcription by the recruitment of the methyltransferase PRMT1, which methylates histone 4 arginine 3 (Rezai-Zadeh et al., 2003).

Moreover, YY1 has been shown to activate transcription by interacting with chromatin remodeling complexes involved in the shifting and repositioning of nucleosomes, such as the INO80 complex (Cai et al., 2007; Wu et al., 2007) and, more recently, the BAF complex (Wang et al., 2018). The interaction with the INO80 complex is also thought to play a role in facilitating the access of YY1 to its target genes (Wu et al., 2007). Hence, through a plethora of molecular co-factor interactions, YY1 influences chromatin modifications and ultimately gene expression.

### YY1 Regulates Transcription Through the 3D Chromatin Organization

At first glance, YY1 does not seem essential for 3D chromatin organization, as the majority of its binding sites are close to transcription start sites (TSSs) and only a minority is located distal from regulated genes (Gabriele et al., 2017; Varum et al., 2019; Zurkirchen et al., 2019). However, in YY1-haploinsufficient lymphoblastoid cell lines, the most differentially expressed genes were controlled by those distal YY1 binding sites (Gabriele et al., 2017). Likewise, in T-helper cells, YY1 seems to mainly influence gene expression through long-distance DNA interactions (Lee, 2014). Interactions between YY1 and proteins involved in chromatin organization, such as CTCF and cohesin (Pan et al., 2013; Schwalie et al., 2013), further implicate a role in 3D chromatin organization.

Loop extrusion allows cohesin to actively form DNA loops (Kim et al., 2019). CTCF, a DNA-binding protein, delimits these DNA loops, demarcating isolated chromatin structures that can be as large as 1 Megabase, called topologically associating domains (TADs) (Zuin et al., 2014; Merkenschlager and Nora, 2016; Rao et al., 2017). Within TADs, long-distance DNA interaction is facilitated between enhancers and promoters by chromatin structuring proteins. CTCF itself is, however, not crucial for the majority of enhancer-promoter interactions (Phillips and Corces, 2009). Recently, YY1 was identified as the structural factor that regulates the formation of DNA loops within the larger CTCF-CTCF domains in a wide variety of mammalian cell types, indicating that YY1-mediated long-distance promoter-enhancer interactions are a general mechanism in mammalian cells (Weintraub et al., 2017). Like CTCF, YY1 forms homodimers and binds hypomethylated DNA to facilitate long-distance DNA interactions. In contrast to CTCF, however, YY1 binds to a consensus sequence mainly present in enhancers and promoters and is only scarcely associates with insulators (López-Perrote et al., 2014; Beagan et al., 2017; Weintraub et al., 2017).

In neuronal differentiation specifically, TAD organization was found to be less conserved between cell types and differentiation stages than initially thought (Wang et al., 2012; Maurano et al., 2015). These findings triggered research into the possible role of dynamic chromatin organization during differentiation of neural progenitor cells (NPCs) (Beagan et al., 2017). Surprisingly, YY1 appeared to instigate DNA loop formation and NPC-specific promoter-enhancer interactions (Beagan et al., 2017). These YY1-mediated DNA loop formations occur within CTCF-CTCF DNA loops (Beagan et al., 2017). These findings introduce a new identity of YY1 as a structural protein in addition to its role as a traditional TF, providing an even broader understanding of the multitude of cellular mechanisms that employ the ubiquitously expressed YY1 protein to regulate transcription.

## YY1 in Health and Disease

### The Role of YY1 in Neurodevelopment

Several studies show that YY1 plays a role in neurodevelopmental disorders (Donohoe et al., 1999; Varum et al., 2019; Zurkirchen et al., 2019), but the mechanisms behind this are still incompletely understood. In mouse embryos, YY1 has proven essential for neurodevelopment (Varum et al., 2019; Zurkirchen et al., 2019; Dong and Kwan, 2020). In murine models, *Yy1* conditional knock-out (cKO) induced at an early stage of cortical development, caused an increased apoptosis rate and cell cycle arrest in neuroepithelium and NPCs (Zurkirchen et al., 2019; Dong and Kwan, 2020). This effect on NPCs however decreased markedly when cKO was induced at later stages (Zurkirchen et al., 2019). Accordingly, another study by Varum and colleagues showed that *Yy1* cKO in mice affects neural crest (NC) development in a strict stage dependent manner. Early KO caused a reduction of multiple NC-derived lineages, whereas late KO (after embryonic day 11.5) resulted in no clear phenotypic difference compared to control (Varum et al., 2019), showing a decreased dependency on YY1 regulated processes at later stages of neuronal development.

Surprisingly, early and late *Yy1* cKO, caused similar changes in gene expression, which indicates that the decreased importance of YY1 during neuronal development does not coincide with a shift in YY1 target genes (Zurkirchen et al., 2019). As YY1-regulated genes seemed to be mainly implicated in metabolic pathways and protein translation, influencing the cell cycle machinery indirectly in NPCs and NC (Varum et al., 2019; Zurkirchen et al., 2019), Zurkirchen and colleagues hypothesized that a decreasing dependency on YY1 during cortical development is due to a decreased biosynthetic demand and decreased proliferation rate of cells at later stages of corticogenesis, making these cells less vulnerable to defects in these pathways.

The importance of YY1 in early development is also attributed to apoptosis inhibition (Sui et al., 2004; Chen et al., 2018; Zurkirchen et al., 2019; Dong and Kwan, 2020). In mice, cKO of *Yy1* at an early embryonic stage caused an accumulation of p53 protein and increased apoptosis (Zurkirchen et al., 2019; Dong and Kwan, 2020). This effect could be partially reversed in *Yy1/Trp53* double cKO mice, indicating that YY1 is important in the downregulation of p53, an important apoptosis regulator, to facilitate NPC survival (Zurkirchen et al., 2019). Additionally, YY1 inhibits apoptosis by regulation of the Planar cell polarity effector gene *FUZ*, an important apoptosis factor in neuronal development. Alterations of *FUZ/Fuz* expression cause neural tube defects in humans and are associated with an increased number and disorganization of NC cells in mice (Gray et al., 2009; Tabler et al., 2013; Seo et al., 2015). YY1 can induce hypermethylation of the *FUZ* promoter, resulting in *FUZ* downregulation and inhibition of its apoptotic signal (Chen et al., 2018). A reduction in soluble YY1 protein reverses this hypermethylation at the *FUZ* promoter and is associated with increased apoptosis (Chen et al., 2018).

Recently, YY1 was also linked to NPC differentiation by downregulation of *Sox2* expression in mice (Knauss et al., 2018). SOX2 is a known pluripotency factor and is also involved in the maintenance of the undifferentiated state of NPCs (Graham et al., 2003). YY1 was implicated in *Sox2* downregulation in mouse brain cortexes during neuronal development by binding the *Sox2* locus and physically halting transcription. These results accentuate a pro-differentiation role for YY1 (Knauss et al., 2018) that contradicts previously described work, which shows that YY1 is vital for NPCs maintenance and proliferation in early development (Varum et al., 2019; Zurkirchen et al., 2019).

Given its complex function, it is not surprising that the role of YY1 in neurodevelopment remains a topic of active discussion. It remains important to also consider YY1 function in a context dependent manner (He and Casaccia-Bonnefil, 2008). Indeed, YY1 has been shown to exert context dependent functions in several cell types, like B-cells and heart muscle (Liu et al., 2007; Beketaev et al., 2015). For neurodevelopment specifically, context dependent YY1 functionality is still incompletely understood and will be an interesting topic to further explore in future studies.

### The Role of YY1 in Disease

In humans, YY1 haploinsufficiency causes Gabriele−de Vries Syndrome, which is characterized by cognitive impairment, behavioral alterations, intrauterine growth restriction, feeding problems and sometimes congenital malformations (Gabriele et al., 2017; Morales-Rosado et al., 2018). One of the consequences of YY1 haploinsufficiency in humans is a loss of H3K27 acetylation at enhancers bound by YY1 (Gabriele et al., 2017). H3K27 acetylation is tightly associated with active promoters and distal enhancers which indicates that downregulation of YY1 affects chromatin regulation and gene transcription (Hebbes et al., 1988; Gabriele et al., 2017). Additionally, mutations in YY1 binding sites in specific non-coding regulatory regions cause neurodevelopmental disorders with a milder phenotype, since the effect of such mutations is limited to the expression of the gene associated with this regulatory region (Gabriele et al., 2018). For example, a disrupted YY1-binding site in a brain-specific enhancer of *ADGRL3* creates a predisposition to attention-deficit/hyperactivity disorder (ADHD) (Martinez et al., 2016).

Throughout life, YY1 regulates various neuroprotective pathways, playing a central role in ischemic damage, Parkinson’s and Alzheimer’s disease. For example, YY1 activates NRF2, which in turn initiates an antioxidant response to protect brain cells against ischemic damage following cerebrovascular accidents (Liu et al., 2018). In Parkinson’s Disease, YY1 is downregulated in microglia, along with other neuroprotective pathways like mTOR and TGF-β (Pal et al., 2016). YY1 regulates the expression of NRF2-mediated anti-oxidant response and the transmembrane transporter SVCT2-dependent import of the protective drug ascorbate (Qiao and May, 2012; Gureev and Popov, 2019), which are key targets for Parkinson’s disease treatment developments. A bioinformatics approach to uncover regulators of Alzheimer’s Disease appointed YY1 as one of the master regulators (Aubry et al., 2015). Interestingly, in contrast to Parkinson’s, in Alzheimer disease higher levels of YY1 mRNA were detected in human autopsy-derived whole brain samples and isolated neuron samples compared to controls (Aubry et al., 2015). It would be tempting to speculate that the protective function requires tightly regulated levels of YY1, while aberrant levels contribute to the onset and progression of neurodegenerative diseases.

Analogous to its role in healthy state, YY1 has a highly context-dependent function also in cancer (Khachigian, 2018; Sarvagalla et al., 2019). YY1 can act as a tumor suppressor or stimulator (Khachigian, 2018). For example, YY1 inhibits proliferation in pancreatic cancer through downregulation of SOX2 (Zhang et al., 2017), while in melanoma, YY1 promotes tumor growth, and YY1 cKO prevents tumorigenesis in a melanoma murine model (Varum et al., 2019). Interestingly, both early NC development and melanoma tumorigenesis seem to be particularly sensitive to alterations in the same metabolic processes that are regulated by YY1 (Varum et al., 2019). Of note, next to YY1, YY2 can also be upregulated in cancer cells (Kaufhold et al., 2017). Possibly, due to the high degree of homology, some YY1 antibodies might not discriminate between the two paralogs, which can be relevant for the interpretation of studies which only employ immunohistochemistry (Kaufhold et al., 2017).

In gliomas, YY1 is overexpressed and contributes to tumor progression (Baritaki et al., 2009). YY1 has been implicated in glioma tumor cell proliferation due to its inhibitory effect on p53 protein levels (Gao et al., 2018). In glioma cell lines, YY1 has been shown to be regulated by the MicroRNA miR-218 (Gao et al., 2018). Normally, miR-218 is downregulated in glioma (Jun et al., 2015). When miR-218 is overexpressed, YY1 is downregulated and the inhibition of the tumor suppressor p53 is nullified (Gao et al., 2018). Additionally, YY1 was shown to upregulate transcription of the long non-coding RNA SNHG17, which activates the Wnt/β-catenin signaling pathway and thus contributes to the proliferation of glioma cells and inhibition of apoptosis (Li et al., 2020).

Proteomics identified YY1 as a regulatory factor involved in cancer stem cell (CSC) maintenance across 17 different cancer types (Kaufhold et al., 2016). YY1 expression was associated with that of CSC regulators SOX2, BMI1, and OCT4. In glioma stem cells (GSCs) Notch signaling was shown to direct YY1 to repress gene transcription involved in cell differentiation by causing locus specific methylation of histone H3K27 (Katsushima et al., 2016). Notch signaling is known to inhibit differentiation and its regulatory mechanisms seem to be vital in neuronal development as well as in GSC maintenance (Bazzoni and Bentivegna, 2019). Together, the ability of YY1 to inhibit apoptosis and its association with CSC maintenance could indicate a broader role for YY1 in tumor biology.

## Concluding Remarks and Future Perspectives

**FIGURE 2:**
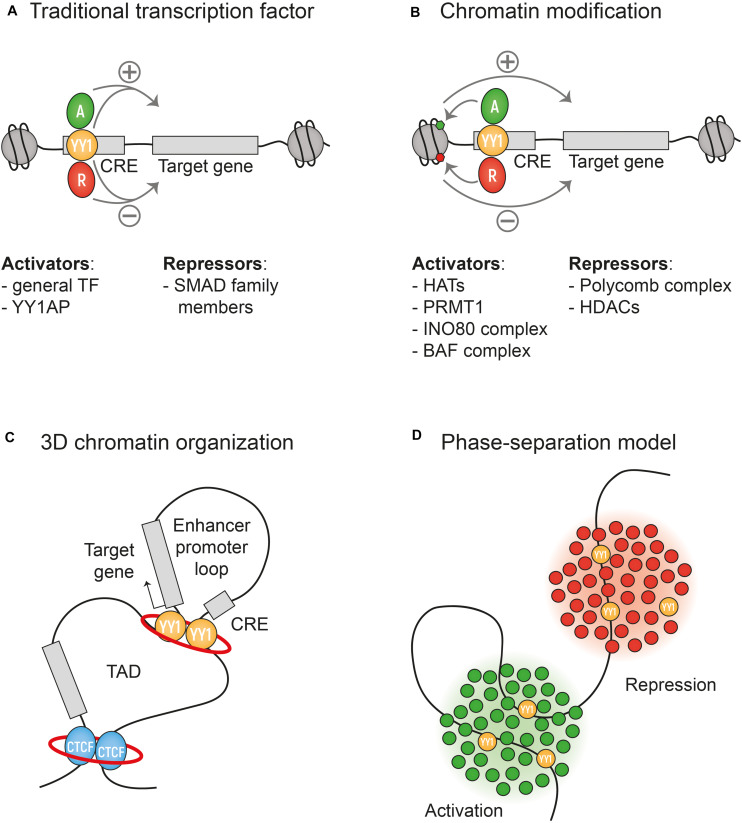
Mechanism of transcriptional activation or repression by YY1. **(A)** YY1 can act as a traditional DNA-binding transcription factor interacting with an extensive list of co-factors that mediate the activation or repression of transcription. Some are listed in the figure. **(B)** YY1 can interact with chromatin remodeling complexes that regulate transcription by regulating chromatin accessibility. **(C)** YY1 regulates transcription via the formation of enhancer-promoter loops within larger TADs. The red ring represents Cohesin. **(D)** The phase separation model might also explain the activity of YY1 as a dynamic activator or repressor of transcription, based on the cocktail of co-factors present in the highly concentrated phase-separated droplets.

YY1 activates or represses genes depending on the cellular context. These seemingly contradictory functions led to the name Yin Yang 1 and sparked investigations into YY1 on a mechanistic level. Here, we briefly discussed the roles that YY1 plays at different levels of transcriptional regulation. First, as a (traditional) DNA-binding TF, YY1 interacts with an extensive list of general and context-dependent cofactors. Recruitment of these cofactors by YY1 is important for direct repression or activation of genes and was shown to play a role in pre-mRNA splicing. Second, YY1 interacts with chromatin remodeling complexes to regulate transcription through chromatin accessibility. Third, by controlling chromatin looping, YY1 can enable and stabilize enhancer-promoter interactions to activate expression (Figure 2).

For the human brain, YY1 is essential for proper development, maintenance, and protection from degeneration. It remains difficult to speculate which of the described roles of YY1 is most important in the brain. As a structural factor, YY1 was shown to facilitate NPC-specific long-distance promoter-enhancer DNA interactions (Beagan et al., 2017). A developmental stage-specific dependency on YY1 in neuronal development, stresses YY1s role in metabolic pathways and protein translation (Varum et al., 2019; Zurkirchen et al., 2019). Further research is needed to elucidate the interplay between YY1 as a regulator of chromatin looping and its role as a traditional TF. Another topic for further research would be the RNA-binding capability of YY1 in relation to transcriptional regulation, as it was shown that RNA binding stabilizes YY1 homodimers and that, compared to DNA binding, the RNA binding occurs with low sequence specificity (Sigova et al., 2015; Wai et al., 2016). In light of the extensive list of YY1 cofactors, it is important to consider the recently proposed phase-separation mechanism, where DNA bound TFs drive the formation of droplets with high concentrations of other TFs and cofactors necessary for transcription (Hahn, 2018; Figure 2D). For example, the TFs OCT4 and GCN4 can form such phase-separated droplets with a high concentration of many cofactors upon enhancer binding (Boija et al., 2018). The high concentration of factors in phase-separated droplets would allow rapid interactions of many factors that during *in vitro* assays would seem too weak (Hahn, 2018). In highly concentrated phase-separated droplets YY1 might be able to interact with even more cofactors than those that have been identified in *in vitro* assays. The ambivalence of YY1 being a repressing and activating TF fits the proposition of the studies on phase-separation that transcriptional regulation is a dynamic biochemical equilibrium (Silveira and Bilodeau, 2018).

Being a ubiquitously expressed transcriptional regulator, it comes as no surprise that YY1 plays a role in multiple regulatory pathways that work in concert to execute the correct transcriptional program in different cell types and at different stages of development. Reviewing the body of literature on YY1, the dualistic reference in its name still fits well. The mechanisms behind seemingly opposite forces of repression and activation are complementary and interconnected. The molecular mechanisms of YY1 also teach us about transcriptional regulation in general. Similar to YY1, other TFs might depend on the cellular context to play different roles with different transcriptional outcomes. GATA1 and co-factor FOG1 for example, activate or repress their target genes in a context-depended manner (Welch et al., 2004), which is mediated by the Nucleosome Remodeling and Deacetylase (NuRD) complex (Miccio et al., 2010). Further research into the different roles of YY1 is warranted. In addition to answering fundamental questions on transcriptional regulation, future studies on YY1 could have clinical implications for neurodevelopmental and neurodegenerative disorders as well as cancer.

## Author Contributions

TV, LH, and EP performed the literature research and wrote sections of the manuscript. TB conceived the manuscript, wrote sections, supervised the work, and obtained funding. All authors approved the final version of the manuscript.

## Conflict of Interest

The authors declare that the research was conducted in the absence of any commercial or financial relationships that could be construed as a potential conflict of interest.
